# Association Study Confirmed Susceptibility Loci with Keloid in the Chinese Han Population

**DOI:** 10.1371/journal.pone.0062377

**Published:** 2013-05-07

**Authors:** Fei Zhu, Baoyu Wu, Ping Li, Jianbo Wang, Huayang Tang, Ye Liu, Xianbo Zuo, Hui Cheng, Yantao Ding, Wen Wang, Yujuan Zhai, Fangfang Qian, Wenju Wang, Xiangfeng Yuan, Jing Wang, Weiwei Ha, Junsheng Hou, Fusheng Zhou, Yin Wang, Jinping Gao, Yujun Sheng, Liangdan Sun, Jianjun Liu, Sen Yang, Xuejun Zhang

**Affiliations:** 1 Institute of Dermatology and Department of Dermatology, No.1 Hospital, Anhui Medical University, Hefei, Anhui, China; 2 Key Laboratory of Dermatology, Anhui Medical University, Ministry of Education, China, Hefei, Anhui, China; 3 State key Laboratory Incubation Base of Dermatology, Anhui Medical University, Hefei, Anhui, China; 4 Department of Plastic Surgery, No.1 Hospital, Anhui Medical University, Hefei, Anhui, China; The Roslin Institute, University of Edinburgh, United Kingdom

## Abstract

Keloid is benign fibroproliferative dermal tumors with unknown etiology. Recently, a genome-wide association study (GWAS) in Japanese population has identified 3 susceptibility loci (rs873549 at 1q41, rs940187 and rs1511412 at 3q22.3, rs8032158 at 15p21.3) for keloid. In order to examine whether these susceptibility loci are associated with keloid in the Chinese Han population, twelve previously reported SNPs were selected for replication in 714 cases and 2,944 controls by using Sequenom MassArray system. We found three SNPs in two regions showed significant association with keloid in the Chinese Han population: 1q41 (rs873549, *P* = 3.03×10^−33^, OR = 2.05, 95% CI: 1.82–2.31 and rs1442440, *P* = 9.85×10^−18^, OR = 0.56, 95% CI: 0.49–0.64, respectively) and 15q21.3 (rs2271289 located in *NEDD4*, *P* = 1.02×10^−11^, OR = 0.66, 95% CI: 0.58–0.74). We also detected one risk haplotype AG (*P* = 1.36×10^−31^, OR = 2.02) and two protective haplotypes of GA and AA (GA, *P* = 1.94×10^−19^, OR = 0.53, AA, *P* = 0.00043, OR = 0.78, respectively) from the two SNPs (rs873549 and rs1442440). Our study confirmed two previously reported loci 1q41 and 15q21.3 for keloid in the Chinese Han population, which suggested the common genetic factor predisposing to the development of keloid shared by the Chinese Han and Japanese populations.

## Introduction

Keloid is a benign, proliferative dermal collagen growth that represents a pathologic wound-healing response to skin injury. It is characterized by an excessive accumulation of extracellular matrix and especially by overabundant collagen formation, which has escaped the boundaries of the original wound to invade the surrounding normal skin and causes aesthetically displeasing and functionally disabling, even leading to the patients to suffer from both physical and psychological distress [1], [2], [3], [4], [5]. Keloid is unique to human and affects some proportion of people in all ethnic populations [1]. Prevalence of keloid varies among different populations, it affects a higher proportion of people of African-Americans and Asians, especially in dark-skinned individuals [6], [7], [8]. There are limited data on Chinese patients with keloid. Several lines of evidence show the importance of genetic factors in keloid [3], [7], [9]. Keloid is more common in ethnicities with darker pigmented skins; the familial heritability and prevalence in twins also support the concept of the genetic predisposition to keloid.

Previous linkage study and candidate gene study have identified genetic factors predisposing to keloid [6], [10], [11], [12], however the results of keloid genetic studies have not been very satisfactory. Recently, GWAS have been proven to be a powerful tool to identify susceptibility genes for common diseases [13]. Nakashima et al [14] performed a GWAS of keloid and identified 3 disease susceptibility loci for keloid in Japanese population. Despite the convincing evidence of its association with keloid in Japanese population, it is not yet known whether these loci play a role in the development of keloid in other populations such as Chinese Han population. The importance of replication in different population should not be overlooked [15].

In this study, we aim to investigate association pattern of these 12 previously reported SNPs for keloid in the Chinese Han population.

## Materials and Methods

### Subjects

A total of 714 patients with keloid and 2,944 controls were recruited consecutively from the outpatients at the Department of Dermatology, NO.1 Hospital, Anhui Medical University. All subjects were of self-reported Chinese Han ancestry (**Table 1**). The clinical diagnosis of all cases was confirmed by at least two dermatologists. Controls were healthy individuals without a diagnosis of keloid, autoimmune and systemic disorders and family history of keloid (including first-, second- and third-degree relatives). All the cases and controls were recruited using uniform criteria and their clinical and demographic information were collected using the same questionnaire. After written informed consent was obtained, peripheral blood samples were collected from all cases and matched healthy controls. The study was approved by the ethical committee of the Anhui Medical University and was conducted according to Declaration of Helsinki principles. DNA was extracted form peripheral blood lymphocytes using QIAamp DNA Blood kit (Qiagen, Valencia, CA, USA) according to the manufacturer’s instructions. The extracted genomic DNAs were analyzed by agarose gel electrophoresis, quantified by spectrophotometer, and stored at −80°C until used.

**Table 1:** Summary information of samples used in replication study.

Characteristic	Case	Control
Total number	714	2944
Gender (male/female)	319/395	1437/1507
Race or ethnicity	Chinese Han	Chinese Han
Age, year, mean ±SD	30.72±12.99	30.38±9.73
Age range, year	2–80	6–70
Family history	–	–

SD, standard deviation.

### SNP Selection and Genotyping

We selected 12 SNPs with at least marginal association evidence (*P*<0.05) based on previous keloid GWAS and other Keloid candidate gene studies and genotyped them in 714 keloid patients. Specifically, 5 SNPs within 3 loci (rs8032158 at *NEDD4*, rs873549 and rs1442440 at 1q41, rs940187 and rs1511412 at 3q22.3, *P*<5.0×10^−8^) and 3 SNPs within 3 loci (rs2271289 at *NEDD4*, rs2983632 at 20p11.21, rs12629284 at 3q23, 5.0×10^−8^<*P*<0.05) based on the Japanese keloid GWAS [14], as well as other four SNPs (rs1866744 at *SMAD6*, rs11071932, rs9806504 and rs2118610 at *SMAD3)* based on Afro-Caribbeans study [16]. MAF for 12 SNPs distribution in CHB and JPT (HapMap data) were showed in **Table 2**. SNPs were genotyped using the Sequenom MassArray system (Sequenom IPLEX assay) at State Key Laboratory Incubation Base of Dermatology, Ministry of National Science and Technology, Hefei, Anhui, China. Approximately 15 ng of genomic DNA was used to genotype each sample. Locus-specific PCR and detection primers were designed using the MassARRAY Assay Design 3.0 software (Sequenom). The DNA samples were amplified by multiplex PCR reactions, and the PCR products were then used for locus-specific single-base extension reaction. The resulting products were desalted and transferred to a 384-element SpectroCHIP array. Allele detection was performed using MALDI-TOF MS. The mass spectrograms were analyzed by the MassARRAY Typer software (Sequenom, San Diego, USA).

**Table 2:** MAF of 12 SNPs distribution in CHB and JPT (Hapmap data).

SNP	Minor allele	MAF	
		CHB	JPT
rs8032158	C	0.357	0.337
rs873549	C	0.333	0.314
rs1442440	C	0.405	0.326
rs2271289	T	0.439	0.401
rs940187	T	0.018	0.087
rs1511412	A	0	0.105
rs11071932	G	0	0
rs1866744	T	0.456	0.489
rs9806504	C	0	0
rs2118610	T	0.137	0.151
rs12629284	T	0.488	0.535
rs2983632	A	0.399	0.43

MAF, minor allele frequency.

### Statistical Analyses

The distributions of MAF for all SNPs in cases and the controls were assessed by Chi-square test and additive model were used for association. Deviation from Hardy–Weinberg equilibrium (HWE) in controls were calculated and all attained *P* values >0.05. Disease associations were analyzed by allelic test, as well as logistic regression and OR and 95% CI were calculated. Independence test of SNPs in the same locus was performed by logistic regression, as well as haplotype-based association test. All statistical analyzes were performed by PLINK 1.07 software [17], unless otherwise specified. Linkage disequilibrium patterns and values were obtained by Haploview v4.2 [18]. Ten SNPs that passed quality control (*P*_HWE_ >0.05 in the control and call rate >90%) were included for further analysis.

## Results

We found significant association evidence at 1q41 (rs873549, *P* = 3.03×10^−33^, OR = 2.05, 95% CI: 1.82–2.31 and rs1442440, *P = *9.85×10^−18^, OR = 0.56, 95% CI: 0.49–0.64, respectively) and 15p21.3 (rs2271289, *P = *1.02×10^−11^, OR = 0.66, 95%CI: 0.58–0.74). The other SNPs did not reach the threshold significant association for keloid (*P*_Bonferroni_ >0.05) in this study. The statistical results of 10 SNPs were summarized in **Table 3**.

**Table 3:** Summary of association results 10 SNPs within 7 loci replicated in the Chinese Han population with keloid.

SNP	Chr	Gene	Allele(minor/major)	MAF		P-value	OR	95% CI
				Case	Control			
rs873549	1q41		G/A	0.53	0.35	3.03×10^−33^	2.05	1.82–2.31
rs1442440	1q41		G/A	0.25	0.37	9.85×10^−18^	0.56	0.49–0.64
rs940187	3q22.3		A/G	0.04	0.03	0.01291	1.48	1.08–2.017
rs1511412	3q22.3	FOXL2	A/G	0.014	0.007	0.01596	1.90	1.12–3.24
rs12629284	3q23		T/C	0.49	0.50	0.5417	0.96	0.86–1.08
rs2271289	15q21.3	NEDD4	T/C	0.35	0.45	1.02×10^−11^	0.66	0.58–0.74
rs1866744	15q22.31	SMAD6	T/C	0.46	0.44	0.2423	1.07	0.95–1.21
rs11071932	15q23	SMAD3	G/A	0.002	0.001	0.194	2.50	0.597–10.48
rs2118610	15q23	SMAD3	A/G	0.114	0.106	0.5168	1.06	0.88–1.28
rs2983632	20p11.21		A/G	0.4076	0.4007	0.6348	1.03	0.91–1.16

MAF, minor allele frequency; OR, odds ratio.

95% CI, 95% confidence intervals.

At 1q41 locus, logistic regression analysis indicated two association signals (rs873549, *P_condition_* = 1.82×10^−16^, OR* = *1.81, rs1442440, *P_condition_* = 0.025, OR = 0.78). Haplotype analysis with rs873549 and rs1442440 showed that significant association evidence for one risk haplotype of AG (*P* = 1.36×10^−31^, OR = 2.02) and two protective haplotypes of GA and AA (GA, *P* = 1.94×10^−19^, OR = 0.53, AA, *P* = 0.00043, OR = 0.78, respectively, **Table 4**).

**Table 4:** Haplotype association analysis between rs873549 and rs1442440 in patients and controls.

rs873549 G/A	rs1442440 G/A	Haplotype	Cases frequency	Controls frequency	OR	P value
G	G	GG	0.019	0.013	1.57	0.1126
A	G	AG	0.506	0.337	2.02	1.36×10^−31^
G	A	GA	0.229	0.356	0.53	1.94×10^−19^
A	A	AA	0.247	0.294	0.78	0.00043

## Discussion

We carried out an association study and confirmed the association of three previously reported SNPs within two susceptibility loci 1q41 (rs873549 and rs1442440) and 15p21.3 (rs2271289) for keloid in the Chinese Han population.

At the locus 1q41, the contributions of rs873549 and rs1442440 were confirmed to show stronger association with keloid in the Chinese population (*P* = 3.03×10^−33^, OR = 2.05, *P = *9.85×10^−18^, OR = 0.56, respectively) than in populations of Japanese ancestry (*P* = 5.89×10^−23^, OR = 1.77, *P* = 8.39×10^−9^, OR = 1.42, respectively) [14]. These findings highlighted that this polymorphic marker showed consistent patterns of genetic contribution to keloid across different racial groups. We observed that the rs873549 (OR = 2.05) and rs1442440 (OR = 0.56) showed risk and protective effect on keloid in the Chinese population, respectively.

In the HapMap database, the SNPs rs873549 and rs1442440 were located at 40-kb LD block, and showed moderately correlated with each other (D’ = 0.95, r^2^ = 0.25, in the Chinese population, and D’ = 0.89, r^2^ = 0.17, in the Japanese population) by linkage disequilibrium (LD) test. Concerning the independent effects of SNPs by logistic regression analysis (rs873549, *P_condition_* = 1.82×10^−16^, OR = 1.81, rs1442440, *P_condition_* = 0.025, OR = 0.78) in our study. Haplotype analysis of the two SNPs (rs873549, rs1442440) showed three haplotypes (AG, GA and AA) had allele frequencies >0.05. The risk haplotype AG had a consistent association evidence for keloid (*P* = 1.36×10^−31^, OR = 2.02) as well as two protective haplotypes of GA and AA (GA, *P* = 1.94×10^−19^, OR = 0.53, AA, *P* = 0.00043, OR = 0.78, respectively). It further supported the presence of two association signals as well as causal variants underlying for keloid within this locus.

Though no reported genes were located within a single 40-kb LD block surrounding the tag SNPs rs873549 and rs1442440 which were associated with keloid in this study. Four no open reading frames of expressed sequence tags (EST) (BG477785, CO245850, BE735115 and BX119652) were found in this region (UCSC database). Nakashima et al [14] confirmed the expression of the two possible non-protein coding genes represented by four ESTs in skin by semiquantitative RT-PCR and not identified any initiation codon recognition sequence or any open reading frames. Hence we should further investigate potential implications of this region for keloid and need functional analysis of these transcripts to clarify their roles on the development of keloid.

At 15p21.3, the SNP rs8032158 within *NEDD4* was significant associated within keloid in populations of Japanese ancestry [14]. In this study, we found rs2271289 located in the intron region of *NEDD4* associated with keloid in the Chinese Han population (*P = *1.02×10^−11^, OR = 0.66), rs8032158 had moderately LD with rs2271289 based on HapMap3 (CHB, D’ = 0.96, r^2^ = 0.41, and JPT, D’ = 1.0, r^2^ = 0.34). The results suggested *NEDD4* that might be a common genetic factor for the development of keloid within multiple populations in terms of Chinese Han and Japanese, although the most significant SNPs were different among them.

Biologically, *NEDD4* is an E3 ubiquitin ligase composed of a C2 domain, three or four WW domains and an ubiquitin ligase Hect domain [19]. *NEDD4* is highly expressed in the skin, skeletal muscle, the liver, the bladder, placenta and cancer cell lines [14], [20]. Previously studies demonstrate that phosphatase and tensin homolog (*PTEN*) [21], insulin-like growth factor I receptor (*IGF-IR*) [22] and *SMAD4* [23] are substrates of *NEDD4*, which have been reported to be associated with keloid. Some studies were indicated *NEDD4* may be involved in cellular proliferation or differentiation through various signaling pathways including *PI3K*, *MAPK* or *TGF-β* [11]. The E3 ubiquitin ligase plays a pivotal role in the *TGF-β* signaling pathway [24], [25]. *NEDD4* was previously suggested to negatively regulate *TGF-β* signaling with ubiquitin-mediated degradation of *SMAD4* [14]. The *TGF-β* family is upregulated in keloid tissue and stimulates the proliferation of fibroblasts. *TGF-β* is also known to promote type I collagen synthesis and inhibit the transcription of collagenase [26]. These facts suggest that the genetic variation(s) in *NEDD4* might affect fibroblast proliferation and keloid formation. However, further study is warranted to explore its exact role in the development of keloid.

At 3p22.3, the SNPs rs940187 and rs1511412 within *FOXL2* were significant associated with keloid in Japanese population (*P* = 1.80×10^−13^, OR = 1.98, and *P* = 2.31×10^−13^, OR = 1.87, respectively) [14]. In this study we did not observed significant association for keloid in Chinese Han population (*P* = 0.013, OR = 1.48, and *P* = 0.016, OR = 1.90, respectively) (P_Bonferroni_ >0.05). OR indicates that these two SNPs probably are associated with keloid in the Chinese Han population. It’s probable that the frequency of these two variants in Chinese Han population was relatively low and the sample size was not very large in this study, therefore the statistical power of association tests was limited. Of course, it also might be due to the existence of susceptibility heterogeneity for keloid between Chinese Han and Japanese populations.

In summary, we not only confirmed two susceptibility loci for keloid (1q41 and *NEDD4* at 15q21.3) in the Chinese Han population but also indicated common genetic factors shared by both Chinese Han and Japanese populations.
